# Absolute quantitative proteomics using the total protein approach to identify novel clinical immunohistochemical markers in renal neoplasms

**DOI:** 10.1186/s12916-021-02071-9

**Published:** 2021-09-06

**Authors:** Susana Jorge, José L. Capelo, William LaFramboise, Swati Satturwar, Dimitrios Korentzelos, Sheldon Bastacky, Gabriela Quiroga-Garza, Rajiv Dhir, Jacek R. Wiśniewski, Carlos Lodeiro, Hugo M. Santos

**Affiliations:** 1 BIOSCOPE Group, LAQV-REQUIMTE, Chemistry Department, NOVA School of Science and Technology, FCT NOVA, Universidade NOVA de Lisboa, 2829-516 Caparica, Portugal; 2 PROTEOMASS Scientific Society, Madan Park, 2829-516 Caparica, Portugal; 3 Department of Pathology, University of Pittsburgh Medical Center, Pittsburgh, PA USA; 4 Biochemical Proteomics Group, Department of Proteomics and Signal Transduction, Max-Planck-Institute of Biochemistry, Martinsried, Germany

**Keywords:** Renal neoplasms, Immunohistochemistry, Mass spectrometry-based proteomics, Total protein approach (TPA), Tissue micro-array (TMA)

## Abstract

**Background:**

Renal neoplasms encompass a variety of malignant and benign tumors, including many with shared characteristics. The diagnosis of these renal neoplasms remains challenging with currently available tools. In this work, we demonstrate the total protein approach (TPA) based on high-resolution mass spectrometry (MS) as a tool to improve the accuracy of renal neoplasm diagnosis.

**Methods:**

Frozen tissue biopsies of human renal tissues [clear cell renal cell carcinoma (*n* = 7), papillary renal cell carcinoma (*n* = 5), chromophobe renal cell carcinoma (*n* = 5), and renal oncocytoma (*n* = 5)] were collected for proteome analysis. Normal adjacent renal tissue (NAT, *n* = 5) was used as a control. Proteins were extracted and digested using trypsin, and the digested proteomes were analyzed by label-free high-resolution MS (nanoLC-ESI-HR-MS/MS). Quantitative analysis was performed by comparison between protein abundances of tumors and NAT specimens, and the label-free and standard-free TPA was used to obtain absolute protein concentrations.

**Results:**

A total of 205 differentially expressed proteins with the potential to distinguish the renal neoplasms were found. Of these proteins, a TPA-based panel of 24, including known and new biomarkers, was selected as the best candidates to differentiate the neoplasms. As proof of concept, the diagnostic potential of PLIN2, TUBB3, LAMP1, and HK1 was validated using semi-quantitative immunohistochemistry with a total of 128 samples assessed on tissue micro-arrays.

**Conclusions:**

We demonstrate the utility of combining high-resolution MS and the TPA as potential new diagnostic tool in the pathology of renal neoplasms. A similar TPA approach may be implemented in any cancer study with solid biopsies.

**Supplementary Information:**

The online version contains supplementary material available at 10.1186/s12916-021-02071-9.

## Background

The diagnosis of oncocytic renal neoplasms is a challenging task with currently available immunohistochemical (IHC) markers. Differential diagnosis includes an eosinophilic variant of clear cell renal cell carcinoma (ccRCC) with the worst prognosis at one extreme and benign renal oncocytoma (RO) at the other extreme [1]. Although some morphological clues help to subclassify these tumors, many oncocytic neoplasms fall under the umbrella of unclassified renal cell carcinomas [2]. The advent of high-resolution mass spectrometry (MS) and advanced software has led to the discovery of novel protein biomarkers for different cancer types including renal carcinomas. However, progress in the discovery of novel biomarkers has been slow with the recent 2016 WHO classification introducing few new entities [3].

MS-based proteomics has become a valuable approach to identify, quantify, and characterize large numbers of proteins in solid and liquid biopsies. The application of MS techniques in determining the presence of amyloid fibril protein in amyloidotic tissues was demonstrated by Gilbertson et al. [4] in a blinded comparison with IHC. Overall, there was significant concordance between the two techniques, but the MS-based approach achieved 94% accuracy, while the diagnostic accuracy of the IHC-based approach was lower at 76%. In MS-based proteomics, label-free approaches are proving advantageous because when labels are not used, there is (i) no limitation to the number of experiments that can be compared, (ii) higher dynamic range of quantification, and (iii) fewer time-consuming steps are needed [5]. Indeed, significant changes can be rapidly measured across an entire proteome and compared in a large cohort of samples. The total protein approach (TPA) is a label-free method that does not require the inclusion of standards either, which measures the absolute amounts of proteins in the sample to deliver large-scale proteomic datasets [6]. TPA has been applied previously to study human colorectal cancer, hepatocyte proteome, and the effects of high-fat diet in mice small intestine mucosa, demonstrating the accuracy and utility of the method [7–9].

In deciphering the molecular landscape of renal cell carcinomas (RCCs), many gel-based MS proteomic studies using label-free quantification (LFQ) have already been reported [10]. One of the early studies that outlined differences in protein levels among different RCC subtypes was carried out by Valera et al. [11] using two-dimensional gel electrophoresis with MS. With advances in MS technology, the conventional gel-based proteomic approaches have been replaced by gel-free strategies, including label-based quantitative studies to evaluate differential expression among proteins [12–14]. High-resolution MS has since been used with LFQ to interrogate the proteome of RCC subtypes [15–17]. The most frequent tumor subtype ccRCC is also the most investigated histological subtype [10]. Despite similarities in the histological features of RCC subtypes, there is extensive molecular heterogeneity among renal neoplasms, and additional biomarkers are needed for comprehensive subtyping.

Advanced-stage renal carcinomas carry a dismal prognosis. Novel methodologies and biomarkers to diagnose these neoplasms in a timely fashion are urgently needed. In this work, we used the TPA to determine the characteristic concentration ranges of a panel of specifically expressed proteins to diagnose different renal neoplasms effectively. A few representative novel biomarkers were validated using IHC on tissue samples.

## Methods

### Study design and sampling

The present work utilized the TPA approach via high-resolution mass spectrometry to analyze the proteomes of 27 flash-frozen, OCT-embedded, human renal tissue biopsies from ccRCC (*n* = 7), papillary renal cell carcinoma (pRCC, *n* = 5), chromophobe renal cell carcinoma (chRCC, *n* = 5), and renal oncocytoma (RO, *n* = 5) and “treatment naïve” normal adjacent tissue (NAT, *n* = 5). To validate the results, IHC analysis was assessed with 128 tissue samples using tissue micro-arrays (TMAs). The samples were collected at the University of Pittsburgh Biospecimen Core and the study was approved by the Institutional Review Board at the University of Pittsburgh (IRB # 02-077). All neoplasms contained a minimum of 85% tumor cells. Data of patients enrolled in this study are summarized in additional file 1: Table S1.

### Proteomic analysis

Biopsies were handled as described in Jorge et al. [18]. Briefly, tissues were first cleaned of optimal cutting temperature (OCT) compound and then proteins were extracted with the aid of an ultrasonic bath (model TI-H-5 from Elma, Singen, Germany) and an ultrasonic probe (UP50H from Hielscher Ultrasonics, Teltow, Germany), respectively. Next, protein digestion was carried out over 4 min using an ultrasonic microplate horn assembly device (QSonica, Newtown, CT, USA). The extracts containing the digested proteomes were subsequently analyzed by a label-free nanoLC-ESI-HR-MS/MS approach (UHR-QqTOF IMPACT HD from Bruker Daltonics, Bremen, Germany).

The mass spectrometry proteomics data have been deposited to the ProteomeXchange Consortium via the PRIDE [19] partner repository with the dataset identifier PXD023296.

### Immunohistochemistry assay using tissue micro-arrays

TMAs were constructed as an orthogonal validation for TPA results using 4 classifications of ccRCC (*n* = 40), pRCC (*n* = 26), chRCC (*n* = 12), RO (*n* = 30), and treatment-naïve normal renal tissue (NAT: *n* = 20). The most differentially expressed proteins for each subtype detected via TPA were selected for validation using TMAs, e.g., perilipin-2 (PLIN2), beta-tubulin III, lysosomal-associated membrane protein-1 (LAMP1), and hexokinase-1 (HK1).

The standard histology deparaffinization protocol was followed for the 4 μm section of paraffin embedded tissue micro-array slides that were used for staining. Different controls included on the TMA slide sections include lung, colon, brain, liver, prostate tissue, and kidney (additional file 2: Fig. S1). Primary antibodies used for IHC include mouse monoclonal antibodies (clone 2C5A3, Abcam) for PLIN2 protein and (clone 2G10, Abcam) for beta-tubulin III and rabbit mono-clonal antibodies (clone EPR10134(B), Abcam) for HK1 protein and (clone EPR4204, Abcam) for LAMP1. Further clone details can be found in additional file 3: Table S2. Antigen retrieval was done using a Decloaking chamber (Biocare Medical, Pacheco, CA) at 120 °C for 2 min with citrate buffer at pH 6.0 (Cell Signaling, Danvers MA). The Envision Dual Link + (DAKO, Carpinteria, CA) was used for detection and visualization was done using 3,3 Diaminobenzidine (DAB) (DAKO). Sections were counterstained with hematoxylin (Cell Signaling) for 5 min, followed by dehydration in ascending alcohol concentrations and xylene followed by glass coverslipping. For PLIN2, membranous or droplet-like staining of any intensity was considered as positive. Percent positivity of tumor cells was scored as (0 = negative; 1:1–10%; 2: > 10–50%; 3: > 50%). Cytoplasmic or membranous staining for beta-tubulin III was considered as positive and scored as (0 = negative; 1:1–10%; 2: > 10–50%; 3: > 50% cells showing positive staining). For HK1 and LAMP1, granular cytoplasmic staining of any intensity was considered as positive. For HK1, granules were scored as (0 = negative, 1 = focal/few, 2 = moderate, 3 = abundant) and for LAMP1 as (0 = negative; D = diffuse, A = apical, F = focal). All TMA cores were scored by one pathologist, and average was taken to give a final score.

The sensitivity, specificity, positive predictive values, and negative predictive values were calculated for IHC biomarkers using a contingency table model (additional file 4: Table S3).

### Data analysis and statistics

Relative label-free quantification was carried out using MaxQuant software V1.6.0.16. All raw files were processed in a single run using defaults settings [20, 21]. Database searches were performed using Andromeda search engine with the UniProt-SwissProt Human database as a reference and a database of common contaminants. Data processing was performed using Perseus V1.6.5.0 with default settings [22, 23]. In brief, reverse hits, and proteins only identified by site were removed from the protein list and normalized spectral protein (label-free quantification/LFQ) intensities were log_2_-transformed to reduce the effect of outliers. Protein groups were filtered based on a minimum presence of 70% in at least one group. Pearson correlation was performed on filtered LFQ values. Missing LFQ values were imputed through generation of random numbers that were drawn from a normal distribution (width = 0.5 and down shift = 1.8). PCA was performed on the filtered and imputed LFQ intensity data. Log ratios were calculated as the difference in average log_2_ LFQ intensity values between the two conditions tested in volcano plots (two-tailed Student’s *t* test, FDR = 0.01 and S0 = 0.1). Differential expression analysis was performed on z-scored log_2_ LFQ intensities through a multiple-sample test (ANOVA test with a 1% of permutation-based FDR filter and preserving randomization for technical replicates). Unsupervised hierarchical clustering was performed based on Euclidean distance.

Absolute protein quantification was calculated using the total protein approach (TPA) using raw spectral intensities from MaxQuant output [24]. Briefly, protein concentration was calculated as follows:

$$ c\ (i)=\frac{\mathrm{MS}\ \mathrm{signal}\ (i)}{\mathrm{total}\ \mathrm{MS}\ \mathrm{signal}\ x\ \mathrm{MW}\ (i)}\ \left[\frac{\mathrm{mol}}{g\ \mathrm{total}\ \mathrm{protein}}\right] $$

## Results

### Renal samples

Solid tumor biopsies were obtained from 22 patients diagnosed respectively with clear cell renal cell carcinoma (ccRCC, *n* = 7), papillary renal cell carcinoma (pRCC, *n* = 5), chromophobe renal cell carcinoma (chRCC, *n* = 5), and renal oncocytoma (RO, *n* = 5) as summarized in additional file 1: Table S1. The proteomes of a total of 27 human tissue specimens, 22 from the diagnosed renal neoplasms described above and 5 from normal adjacent renal tissue (NAT), were interrogated by high-resolution MS. The NAT samples (*n* = 5) were used as controls to determine the extent and profile of protein expression deregulation in each tumor subtype.

### Proteomics analysis

**Fig. 1:**
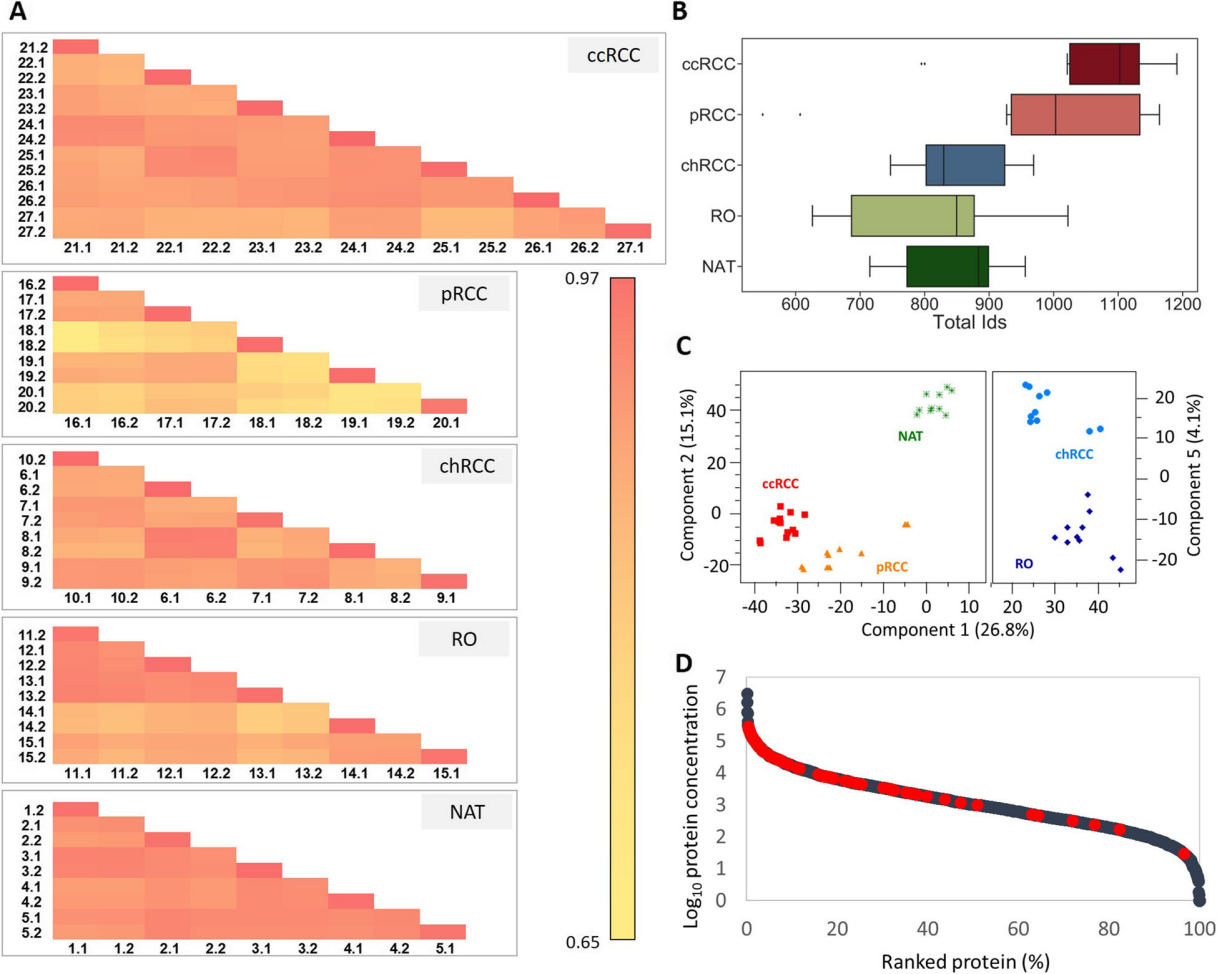
Statistical analysis of renal tissue proteome data. **A** Pearson correlation coefficients between biological and technical replicates for each tumor subtype and the normal adjacent tissue (NAT) control. For example, 1.1 and 1.2 correspond to sample 1 replicate 1 and replicate 2, respectively. **B** Numbers of proteins identified for each tumor subtype and NAT. **C** Principal component analysis using the set of 1234 proteins selected for quantification (see text for details). **D** Distribution of TPA-based protein concentrations (pmol of protein per mg of total protein) highlighting proteins described in the literature as potential ccRCC markers (red dots, proteins are listed in additional file 6: Table S5). Subtypes: ccRCC, clear cell renal cell carcinoma; pRCC, papillary renal cell carcinoma; chRCC, chromophobe renal cell carcinoma; RO, renal oncocytoma

Liquid chromatography coupled to tandem mass spectrometry (nanoLC-ESI-HR-MS/MS) was used to analyze the tissue biopsies in duplicate resulting in 54 LC-MS/MS runs. The correlation between readouts from technical and biological replicates in each sample group was tabulated, an indication of the level of reproducibility (Fig. 1A). The Pearson correlation coefficients ranged from 0.65 to 0.97. It is noteworthy that the correlation coefficients of technical replicates (for the LC-ESI-HR-MS/MS step) are higher than 0.94 for all the tumor subtypes studied. In terms of biological variability (between samples of the same subtype), the pRCC samples were the least homogeneous, which is consistent with the well-known characteristics of this cancer subtype [25]. A total of 2547 proteins were identified across all tissue biopsies and the statistics summarizing the number of proteins identified in each tumor subtype are shown in Fig. 1B. To ensure the robustness of the quantification method, only the identified proteins with a reproducibility higher than 70% in at least one tumor subtype were considered for further analysis. A set of 1234 proteins fulfilled this criterion, and they were used in the following quantification step.

### Protein quantification

Relative quantification of the selected set of 1234 proteins was done by LFQ using MaxQuant software [21]. The LFQ values of the dataset of proteins are summarized in additional file 5: Table S6. Principal component analysis (PCA) of the data from all samples was done to find possible correlation (Fig. 1C). According to the principal components underlying the variation in the data, all tumor proteomes are clearly differentiated from the NAT proteome and from other RCC subtypes.

Absolute amounts of proteins were calculated based on raw intensities in the mass spectra using the TPA method [24]*.* The dynamic range of protein abundances measured in this study span approximately seven orders of magnitude (Fig. 1D). This is consistent with previous studies using the TPA approach to interrogate solid biopsies [7].

### Label-free MS-based protein profiles

**Fig. 2:**
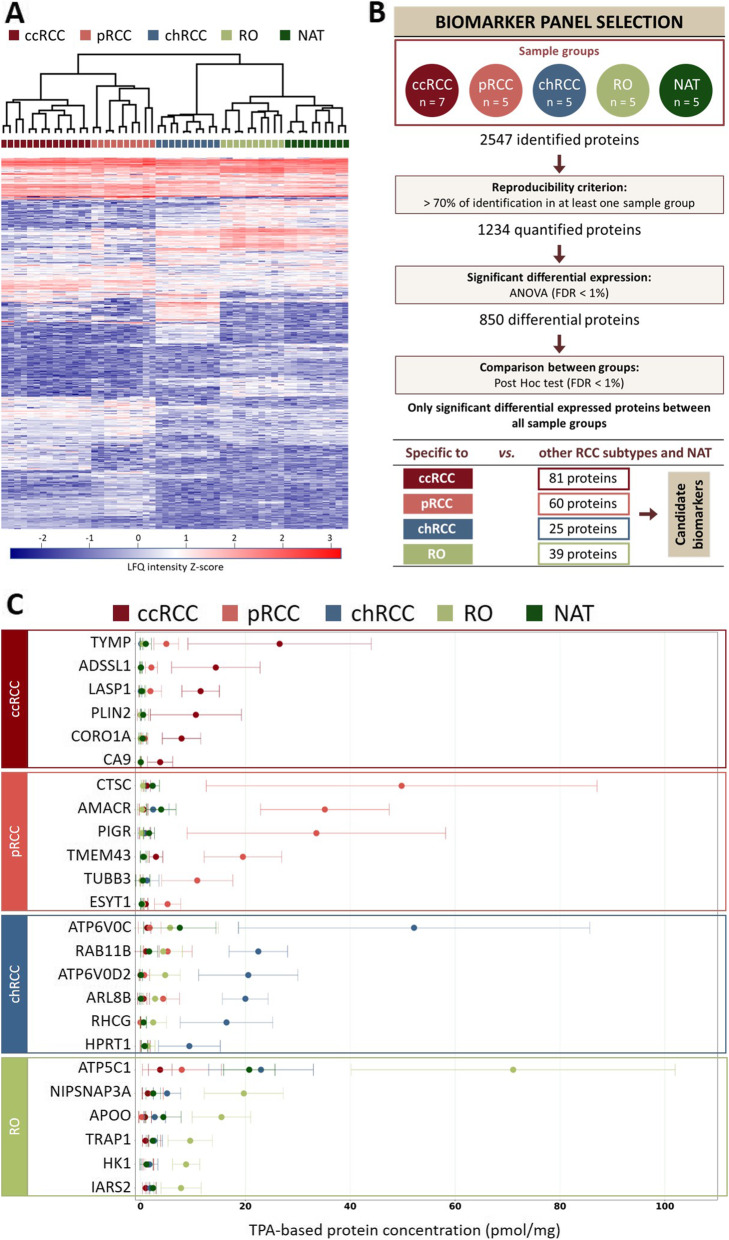
Statistically significant differential expression of proteins in renal neoplasms. **A** Hierarchical clustering analysis of 850 differentially expressed proteins (ANOVA, FDR < 1%) according to the tissue source. **B** Statistical proteomic workflow to select panels of proteins to discriminate between the four renal neoplasm subtypes and normal adjacent tissue (NAT). **C** TPA-based concentrations of the proteins with the highest differential expression between tissue biopsies (fold change) for each subtype. Clear cell renal cell carcinoma (ccRCC, FC ≥ 5); papillary renal cell carcinoma (pRCC, FC ≥ 9); chromophobe renal cell carcinoma (chRCC, FC ≥ 4); renal oncocytoma (RO, FC ≥ 3), additional file 7: Fig. S2. Absolute protein concentration expressed in pmol/mg of total protein was calculated with the TPA method [24]

To assess which of the selected set of 1234 proteins are significantly upregulated or downregulated in each tumor type, a multi-sample test (ANOVA with a filter for permutation-based false detection rate < 1%) was applied. Expression levels of 850 proteins were found to be statistically different between sample groups. As shown in Fig. 2A, the unsupervised clustering analysis performed on differentially expressed proteins clearly divided the samples into two groups, one comprising ccRCC and pRCC and the other comprising chRCC, RO and NAT.

Among the 850 proteins defined as being differentially expressed, biomarkers were sought that could potentially be used to differentiate between each tumor type. A schematic representation of the workflow for candidate biomarker selection is shown in Fig. 2B. The comparison of protein abundances between sample groups revealed a set of 205 proteins that distinguished each tumor subtype (ANOVA, *p* < 0.01). These proteins form potential biomarker panels comprising 81 proteins for ccRCC, 60 for pRCC, 25 for chRCC, and 39 for RO (additional file 5: Table S4).

### TPA-based concentration range of diagnostic proteins

Next, the TPA method was used to transform the raw spectral intensity values of the four protein panels (Fig. 2B), converting them into absolute concentration values (additional file 6: Table S5). For each tumor subtype, we selected the 6 proteins with the largest differences in concentration levels (Fig. 2C).

### Evaluating the TPA-based results against published data

As proof of concept, unique proteins identified in ccRCC using the TPA-based method were compared with proteins already described in the literature as characterizing ccRCC. Approximately 90 proteins have been described as putative diagnostic markers of the ccRCC subtypes in the current literature, and 46 of them were detected in our analysis (Table 1). Other proteins described as putative biomarkers in the literature were also found in our intermediate datasets, but the differential expression of those proteins was not considered to be statistically significant according to our threshold.

**Table 1:** Protein list of common deregulated proteins achieved in literature and our data

Protein name	Gene name	ccRCC/NAT expression	Ref.
Acetyl-CoA acetyltransferase, mitochondrial	ACAT1	Down	[16]
Alcohol dehydrogenase [NADP(+)]	AKR1A1	Down	[26]
Retinal dehydrogenase 1	ALDH1A1	Up	[26]
Fructose-bisphosphate aldolase	ALDOA	Up	[17]
Aminopeptidase N	ANPEP	Down	[27]
Annexin A4	ANXA4	Up	[28]
Annexin A5	ANXA5	Up	[26]
Aquaporin-1	AQP1	Down	[29]
Carbonic anhydrase 9	CA9	Up	[30]
Calbindin	CALB1	Down	[26, 31]
Macrophage-capping protein	CAPG	Up	[32]
Cofilin-1	CFL1	Up	[17]
Coronin-1A	CORO1A	Up	[15]
Dipeptidase 1	DPEP1	Down	[29]
Alpha-enolase	ENO1	Up	[11, 14, 33]
Gamma-enolase	ENO2	Up	[26, 32]
Fatty acid-binding protein, brain	FABP7	Up	[34, 35]
Gelsolin	GSN	Up	[31]
Glutathione S-transferase P	GSTP1	Up	[26]
Heat shock protein beta-1	HSPB1	Up	[11, 14, 32]
Plastin-2	LCP1	Up	[32]
L-lactate dehydrogenase A chain	LDHA	Up	[12, 14]
Galectin-1	LGALS1	Up	[13, 34]
Major vault protein	MVP	Up	[12]
Nucleoside diphosphate kinase A	NME1	Up	[32]
Nicotinamide N-methyltransferase	NNMT	Up	[12, 17, 32, 36]
Profilin-1	PFN1	Up	[13, 17]
Pyruvate kinase PKM	PKM	Up	[37, 38]
Perilipin-2	PLIN2	Up	[12, 15, 39]
Peptidyl-prolyl cis-trans isomerase A	PPIA	Up	[34]
Peroxiredoxin-4	PRDX4	Up	[32]
UV excision repair protein RAD23 homolog B	RAD23B	Up	[11]
Histone-binding protein RBBP7	RBBP7	Up	[32]
Reticulocalbin-1	RCN1	Up	[40]
Protein S100-A10	S100A10	Up	[41]
Protein S100-A11	S100A11	Up	[41, 42]
Plasma protease C1 inhibitor	SERPING1	Up	[12]
Serpin H1	SERPINH1	Up	[43]
ADP/ATP translocase 3	SLC25A6	Down	[26]
Protein-glutamine gamma-glutamyltransferase 2	TGM2	Up	[26]
Triosephosphate isomerase	TPI1	Up	[11]
Tubulin alpha-1B chain	TUBA1B	Up	[32]
Thymidine phosphorylase	TYMP	Up	[12, 32]
Vimentin	VIM	Up	[26, 33, 44];
14-3-3 protein zeta/delta	YWHAZ	Up	[13]

**Fig. 3:**
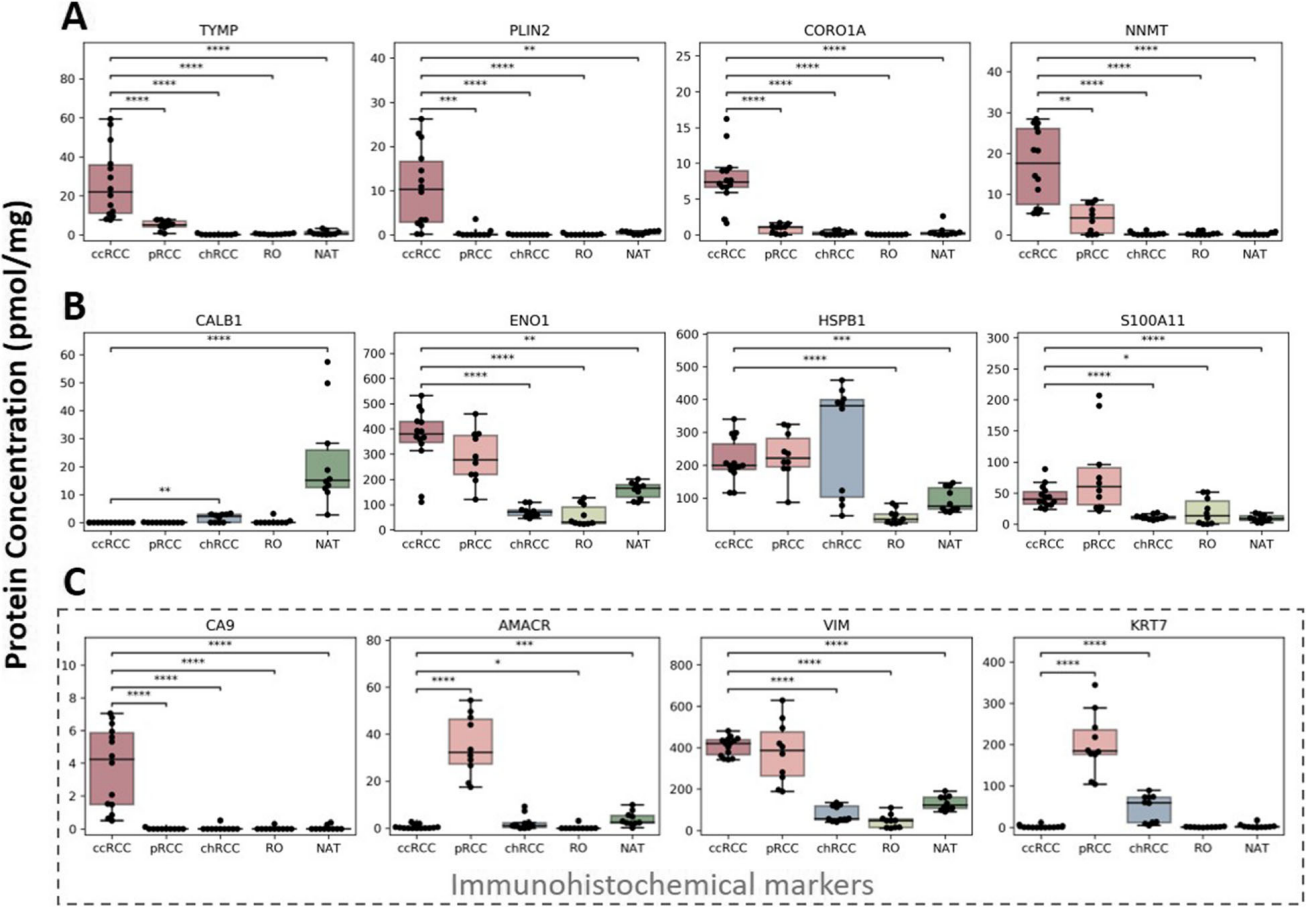
Renal tissue protein concentrations of putative diagnostic markers measured by TPA approach. **A** Candidate biomarkers to distinguish subtype ccRCC from other subtypes and normal adjacent tissue (NAT). **B** Candidate biomarkers to distinguish ccRCC from NAT but not from other subtypes. **C** Proteins widely used in immunohistochemical diagnosis of renal carcinomas. Statistical analysis was performed using the pairwise Mann-Whitney test (*p ≤ 0.005; **p ≤ 0.001; ***p ≤ 0.0001; ****p ≤ 0.00001). Subtypes: ccRCC, clear cell renal cell carcinoma; pRCC, papillary renal cell carcinoma; chRCC, chromophobe renal cell carcinoma; RO, renal oncocytoma

Statistical analysis on 12 of the 46 proteins is presented here (Fig. 3), but all 46 were analyzed in the same way (additional file 8 Fig. S3 and additional file 9: Fig S4). The 12 proteins are presented in three groups to demonstrate their discriminatory potential. First, TYMP, PLIN2, CORO1A, and NNMT are biomarkers that can differentiate ccRCC from all other subtypes and NAT (Fig. 3A). Second, candidate proteins to distinguish ccRCC from NAT but not necessarily from other subtypes are CALB1, ENO1, HSPB1, and S100A11 (Fig. 3B). The reported concentrations of the most widely used IHC markers to diagnose and distinguish between ccRCC and pRCC, namely CA9, AMACR, VIM, and KRT7, are also presented for comparison (Fig. 3C).

### IHC validation of four TPA-derived markers for renal neoplasm subtypes

**Fig. 4:**
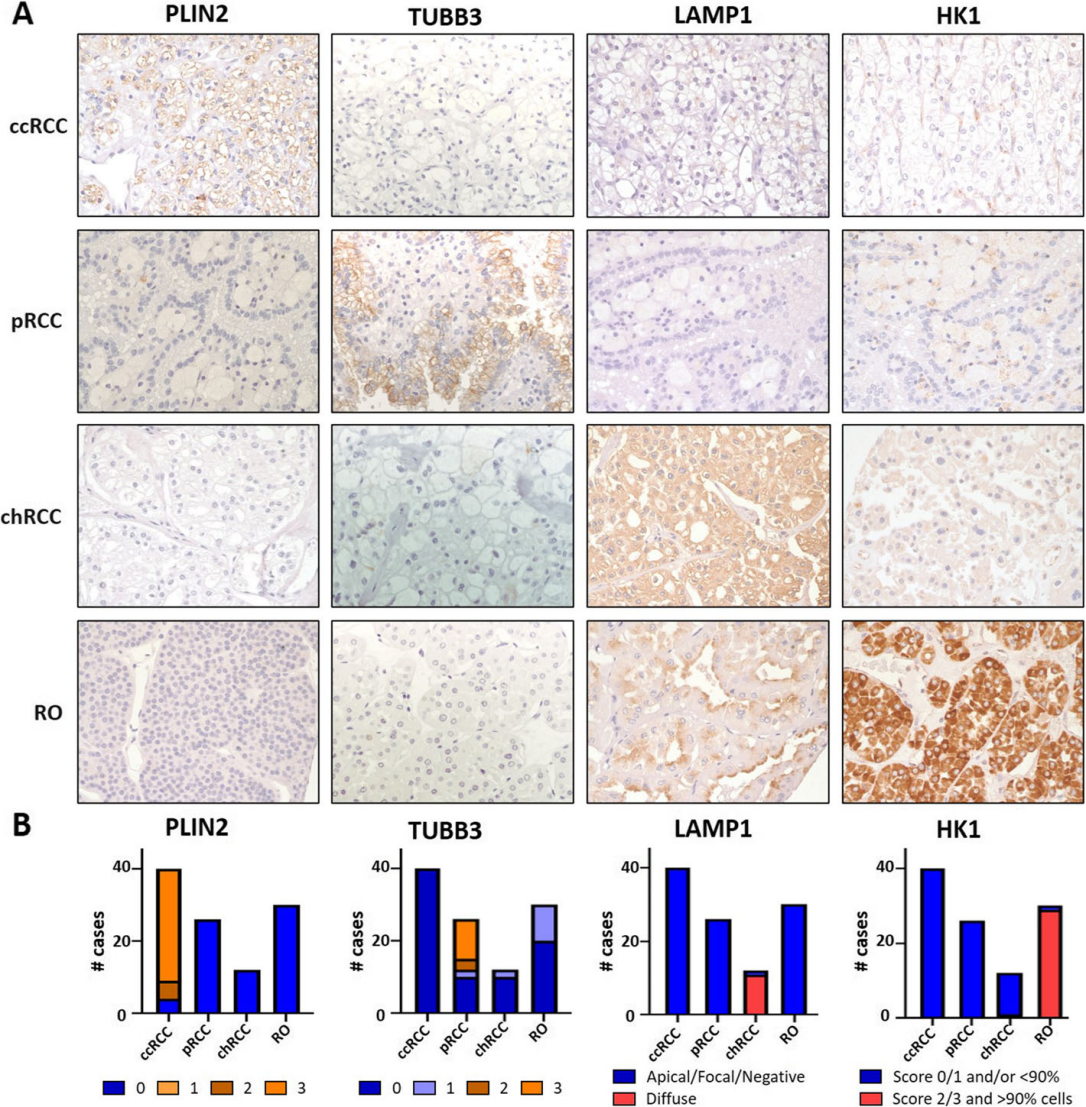
Immunohistochemical staining of renal neoplasms with four markers identified by TPA. **A** Staining of perilipin 2 (PLIN2), beta-tubulin III (TUBB3), lysosomal associated membrane protein 1 (LAMP1), and hexokinase 1 (HK1) in renal neoplasm subtypes. **B** Bar graphs representing the number of cases and extent of staining for PLIN2, TUBB3, LAMP1, and HK1. For PLIN2 and TUBB3, the percentage of tumor cells that stained positive was scored as 0 for negative, 1 for 1–10%, 2 for > 10–50%, and 3 for > 50%. For HK1, the extent of staining was scored as 0 for negative, 1 for focal or few cells, 2 for moderate, and 3 for abundant. Subtype abbreviations: ccRCC, clear cell renal cell carcinoma; pRCC, papillary renal cell carcinoma; chRCC, chromophobe renal cell carcinoma; RO, renal oncocytoma

Both the TPA and datamining results indicated that perilipin-2 (PLIN2) was a potential biomarker to distinguish ccRCC from pRCC, chRCC, and RO (Fig. 2C and Fig. 3A). When an IHC method was used to test for the presence of PLIN2 on renal TMAs, the majority of the ccRCC samples tested were positive for PLIN2 whereas pRCC, chRCC, and RO samples were negative (Fig. 4). Benign kidney tissue was also negative for PLIN2 (data not shown). When PLIN2 stained more than 10% of cells in a sample (scored as 2 or 3), 90% of ccRCC cases were detected with a 100% specificity distinguishing ccRCC from other renal neoplasms. By comparison, beta-tubulin III (TUBB3), a potential biomarker for pRCC (Fig. 2C), showed variable IHC staining in pRCC with half of cases being more than 10% positive, but staining was negative in all ccRCC and less than 10% positive in all chRCC and RO. Benign kidney tissue showed focal staining of TUBB3 in tubules (data not shown). By setting a staining score cutoff > 2 (equivalent to > 10% of cells staining positive) for TUBB3, this biomarker has a sensitivity of 53.8% and a specificity of 100% in distinguishing pRCC from other renal neoplasms (Fig. 4). Staining for lysosomal associated membrane protein 1 (LAMP1), a candidate in the chRCC biomarker panel, was diffuse in chRCCs, apical or focal in ROs, and negative in other subtypes. Diffuse LAMP1 staining thus has a sensitivity of 91.7% and specificity of 100% to distinguish chRCC from other renal neoplasms. IHC staining for hexokinase 1 (HK1) was positive and diffuse in most ROs (Fig. 2C and Fig. 4). By setting a cutoff score above 2 and > 90% cell positivity, HK1 has a sensitivity of 96.7% as a biomarker with a specificity of 98.7% in distinguishing ROs from other renal neoplasms (Fig. 4). Both HK1 and LAMP1 markers showed variable positivity in normal kidney tubules (data not shown) and were negative in most ccRCCs and pRCCs (Fig. 4).

## Discussion

Timely diagnosis is needed for early intervention in treating patients with advanced-stage renal carcinomas. TNM stage, tumor subtype, and grade are among the most important prognostic factors. The absence of comprehensive prognostic biomarkers limits current diagnostic and prognostic models emphasizing the need to validate novel diagnostic, prognostic, and therapeutic biomarkers for renal neoplasms [45]. There is increasing evidence that renal neoplasms represent a group of histologically and molecularly heterogeneous diseases, even within the same histological subtype [46]. Many molecular markers identified in different renal neoplasms currently lack validation studies to allow their use in routine diagnostic use. Here, we interrogated 27 renal proteomes comprising four common histological neoplasm subtypes using the TPA method.

Our study showed that MS data obtained with LFQ in biological and technical replicates was of excellent quality, with the similarity of technical replicates exceeding 97%. Our data indicated that pRCC proteomes presented the highest biological variability between biopsies, while NAT proteomes were the most homogeneous as expected. It is indeed recognized that pRCC displays a morphological pattern shared by several different types of renal cell carcinomas [47]. The PCA and cluster analysis indicated that the proteomes of the ccRCC and pRCC subtypes are more similar to each other than to either the chRCC or RO proteomes (Fig. 1C and Fig. 2A). These results likely reflect the different cellular origins of ccRCC and pRCC which originate from proximal tubule cells, while chRCC and RO originate from intercalated cells of the distal nephron and collecting ducts [48]. Interestingly, the benign subtype, RO, more closely resembled NAT than any other subtype, consistent with its clinical outcome (Fig. 2A).

The use of LFQ-based values from MS results allowed us to identify a total of 850 proteins that are differentially expressed between groups. Further analyses of these 850 proteins, using the TPA-based concentrations and statistics, narrowed down a number of proteins that specifically identify each renal neoplasm subtype versus all other subtypes and NAT. Large sets of protein panels present in all tumor subtypes discriminate each tumor subtype because the range of concentrations of these proteins are unique, and this information can be used to classify biopsies.

As a proof of concept, we have compared the 81 proteins found as unique for ccRCC with those reported in the literature. Remarkably, 46 of our suggested biomarkers have been described previously. For instance, thymidine phosphorylase (TYMP), PLIN2 and coronin-1A (CORO1A) proteins have been proposed as putative markers for ccRCC. TYMP protein is associated with pro-angiogenic and anti-apoptotic effects in cancer cells [49, 50], and higher levels of TYMP in RCC tissue versus non-neoplastic kidney tissues have also been described [51]. Our results showed TYMP to be one of the most up-regulated proteins in ccRCC. The same was true for PLIN2, which is known to be highly expressed in the clear cells of ccRCC [12, 15, 39]. PLIN2 regulates lipid metabolism and storage and positively correlates with HIF-2α which drives cell proliferation and survival [52, 53]. The levels of COROA1 protein were also found exclusively upregulated in ccRCC. COROA1 is crucial for cytoskeleton modulation [54]. High levels of this protein have been reported in a variety of renal neoplasms within tumor-infiltrating lymphocytes [15].

IHC markers routinely used for the diagnosis of renal neoplasms are summarized in additional file 10: Table S6 [55–57]. For example, carbonic anhydrase 9 (CA9) is one of the most sensitive and specific IHC markers for ccRCC while racemase/AMACR is used routinely to detect pRCC. Similarly, our proteomic data showed high levels of CA9 protein in ccRCC and high AMACR levels in pRCC. We also identified cytokeratin 7 (KRT7) to be prominently expressed in pRCC. Vimentin (VIM) immunostaining is generally positive in ccRCC (+) and pRCC (+/−) and negative in chRCC and RO. Our approach confirmed these findings with VIM protein levels elevated in ccRCC and pRCC versus NAT while in chRCC they were slightly lower than in NAT.

Semi-quantitative IHC validation of the biomarkers using TMAs corroborated our proteomic data. One of the novel biomarkers was PLIN2 which was positive in a majority of the ccRCC tested. We found TUBB3 to be a specific marker for pRCC, albeit with a low sensitivity. TUBB3 is a component of microtubules and is involved in mitosis, cell motility and intra-cellular transport. It is expressed in normal testis and neural crest derived tissue and is considered a prognostic biomarker as it is associated with aggressive tumor behavior in ovarian and other carcinomas [58, 59]. It should be noted that most of the TUBB3 that were positive for pRCC in our TMAs were scored independently as having a WHO 2016/ISUP nuclear grade of 2 or more. Diffuse LAMP1 positivity was very specific for chRCC in our validation cohort, similar to the findings of Drendel et al. [60]. Interestingly, our results showed that HK1 was positive in most oncocytomas, but negative in other renal neoplasms. HK1 is an enzyme involved in the glycolysis pathway and it is located at the outer membrane of mitochondria. Studies have shown that HK1 is involved in regulating cell death, carcinogenesis, and cell proliferation [61, 62].

To summarize, the TMA results suggest that PLIN2 may serve as a sensitive and specific marker for ccRCC, beta-tubulin III for pRCC, HK1 for RO, and diffuse LAMP1 for chRCC. Although PLIN2 plays a role in lipid and phospholipid storage and HK1 in the glycolysis pathway, their specific roles in renal carcinogenesis remain to be elucidated. In addition to its diagnostic potential, TUBB3 may emerge as a prognostic marker for pRCC.

## Conclusions

In conclusion, our study explores the utility of the novel TPA method in the differential diagnosis of multiple renal neoplasm subtypes. TPA-based pathology may provide a new approach to complement and extend the conventional histological diagnosis paradigm. This high-throughput technology can be used to quantify hundreds of proteins at the same time, thus enabling rapid and reproducible screening and subtyping of renal neoplasms. As an indication, the assays for this study spanned just two weeks. TPA-based technology represents a versatile tool for quick identification of new biomarkers that may impact diagnosis, prognosis, and therapeutic guidance when applied to renal tumors. Although the number of tissues used to obtain the proteins levels with which the candidate proteins were found is 27, the number of qualitative immunochemistry assays used to validate them, 128, was statistically significant to confirm the validity of this study. Future studies will be required to validate TPA-based pathology on prospective cohorts to assess its performance as a frontline diagnostic tool, which is part of ongoing efforts.

## Supplementary Information

**Additional file 1: Table S1.** Patient’s data.

**Additional file 2: Fig. S1.** Different controls included on the TMA slide sections include lung, nerve, brain tissue, and kidney.

**Additional file 3: Table S2.** Details of antibodies used for validation immunohistochemistry.

**Additional file 4: Table S3.** Sensitivity, specificity, positive predictive values, and negative predictive values calculated for IHC biomarkers.

**Additional file 5: Table S4.** LFQ values of proteins and significant differential expressed proteins.

**Additional file 6: Table S5.** TPA concentration values of significant differential expressed proteins.

**Additional file 7: Fig. S2.** TPA-based concentrations of the proteins with the highest differential expression between tissue biopsies (fold change) for each subtype. Clear cell renal cell carcinoma (ccRCC, FC ≥ 5); papillary renal cell carcinoma (pRCC, FC ≥ 9); chromophobe renal cell carcinoma (chRCC, FC ≥ 4); renal oncocytoma (RO, FC ≥ 3).

**Additional file 8: Fig. S3.** TPA concentration values of proteins described in literature - Part I. Statistical analysis was performed using pairwise Mann Whitney test (*p ≤ 0.005; **p ≤ 0.001; ***p ≤ 0.0001; ****p ≤ 0.00001).

**Additional file 9: Fig. S4.** TPA concentration values of proteins described in literature - Part II. Statistical analysis was performed using pairwise Mann Whitney test (*p ≤ 0.005; **p ≤ 0.001; ***p ≤ 0.0001; ****p ≤ 0.00001).

**Additional file 10: Table S6.** Current available IHC markers for the diagnosis of renal neoplasms.

## Data Availability

The mass spectrometry proteomics data have been deposited to the ProteomeXchange Consortium via the PRIDE [19] partner repository with the dataset identifier PXD023296.

## Abbreviations

CA9: Carbonic anhydrase 9
ccRCC: Clear cell renal cell carcinoma
chRCC: Chromophobe renal cell carcinoma
CORO1A: Coronin-1A
FDR: False discovery rate
HK1: Hexokinase-1
IHC: Immunohistochemical
KRT7: Cytokeratin 7
LAMP1: Lysosomal associated membrane protein-1
LFQ: Label free quantification
MS: Mass spectrometry
NAT: Normal adjacent tissue
OCT: Optimal cutting temperature
PCA: Principal component analysis
PLIN2: Perilipin-2
pRCC: Papillary renal cell carcinoma
RCC: Renal cell carcinoma
RO: Renal oncocytoma
TMA: Tissue micro-arrays
TPA: Total protein approach
TUBB3: beta-Tubulin III
TYMP: Thymidine phosphorylase
VIM: Vimentin
