# Supplementary material for: Absolute quantitative proteomics using the total protein approach to identify novel clinical immunohistochemical markers in renal neoplasms
Source: BMC Med. 2021 Sep 6;19:196. doi: 10.1186/s12916-021-02071-9 (PMC8420025; doi:10.1186/s12916-021-02071-9)
Supplement: Supplementary file 1 — Additional file 1: Table S1. Patient’s data. [file 12916_2021_2071_MOESM1_ESM.docx]

**Table S1.** Description of human kidney biopsies used in the study.

| BIOPSY | AGE | GENDER | DIAGNOSIS | SAMPLE TYPE |
| --- | --- | --- | --- | --- |
| N1 | 54 | Male | RCC | NAT |
| N2 | 49 | Female | Papillary | NAT |
| N3 | 58 | Female | RCC | NAT |
| N4 | 72 | Female | RCC | NAT |
| N5 | 70 | Male | RCC | NAT |
| C6 | 73 | Male | RCC | chRCC |
| C7 | 67 | Female | RCC | chRCC |
| C8 | 71 | Male | RCC | chRCC |
| C9 | 58 | Female | RCC | chRCC |
| C10 | 81 | Male | RCC | chRCC |
| O11 | 80 | Male | RCC | RO |
| O12 | 69 | Female | RCC | RO |
| O13 | 63 | Male | RCC | RO |
| O14 | 62 | Female | RCC | RO |
| O15 | 55 | Female | RCC | RO |
| P16 | 51 | Female | RCC | pRCC |
| P17 | 66 | Male | RCC | pRCC |
| P18 | 75 | Male | RCC | pRCC |
| P19 | 87 | Male | RCC | pRCC |
| P20 | 69 | Male | RCC | pRCC |
| CC21 | 50 | Male | RCC | ccRCC |
| CC22 | 68 | Male | RCC | ccRCC |
| CC23 | 60 | Male | RCC | ccRCC |
| CC24 | 68 | Female | RCC | ccRCC |
| CC25 | 58 | Male | RCC | ccRCC |
| CC26 | 42 | Female | RCC | ccRCC |
| CC27 | 58 | Female | RCC | ccRCC |

RCC: renal cell carcinoma; NAT: normal adjacent tissue; chRCC: chromophobe renal cell carcinoma; RO: renal oncocytoma; pRCC: papillary renal cell carcinoma; ccRCC: clear cell renal cell carcinoma.
