# Supplementary material for: Absolute quantitative proteomics using the total protein approach to identify novel clinical immunohistochemical markers in renal neoplasms
Source: BMC Med. 2021 Sep 6;19:196. doi: 10.1186/s12916-021-02071-9 (PMC8420025; doi:10.1186/s12916-021-02071-9)
Supplement: Supplementary file 2 — Additional file 2: Fig. S1. Different controls included on the TMA slide sections include lung, nerve, brain tissue, and kidney. [file 12916_2021_2071_MOESM2_ESM.docx]

**Table S2.** Details of antibodies used for validation immunohistochemistry

| **Antibody** | **Clone** | **Dilution** | **Antigen Retrieval** | **Vendor** | **Positive Control** | **Negative control** |
| --- | --- | --- | --- | --- | --- | --- |
| **Perilipin 2 (PLIN 2)** | 2C5A3 | 1:200 | Citrate buffer | Abcam | Lung and others | Brain |
| **Beta tubulin III** | 2G10 | 1:200 | Citrate buffer | Abcam | Nerves in normal kidney | Lung |
| **Hexokinase 1 (HK-1)** | EPR10134(B) | 1:100 | Citrate buffer | Abcam | Kidney & others | #Not applicable |
| **Lysosomal associated membrane protein 1 (LAMP-1)** | EPR4204 | 1:100 | Citrate buffer | Abcam | Kidney & others | #Not applicable |

# In general LAMP-1 and HK-1 shows at least focal granular positivity in all tissue types thus precluding negative control tissue.

**IHC method details:**

The standard histology deparaffinization protocol was followed for the 4 µm section of paraffin embedded tissue micro-array slides that were used for staining. Different controls included on the TMA slide sections include lung, colon, brain, liver, prostate tissue, and kidney. The primary antibodies, perilipin-2, beta tubulin III, hexokinase-1, Lysosomal associated membrane protein 1(LAMP-1) were utilized. (Clone details Table 5). Antigen retrieval was done using a Decloaking chamber (Biocare Medical, Pacheco, CA) at 120°C for 2 minutes with citrate buffer at pH6.0 (Cell Signaling, Danvers MA). The Envision Dual Link + (DAKO, Carpinteria, CA) was used for detection and visualization was done using 3,3 Diaminobenzidine (DAB) (DAKO). Sections were counterstained with hematoxylin (Cell Signaling) for 5 minutes, followed by dehydration in ascending alcohol concentrations and xylene followed by glass coverslipping.
