# Supplementary figures and images for: Absolute quantitative proteomics using the total protein approach to identify novel clinical immunohistochemical markers in renal neoplasms
Source: BMC Med. 2021 Sep 6;19:196. doi: 10.1186/s12916-021-02071-9 (PMC8420025; doi:10.1186/s12916-021-02071-9)

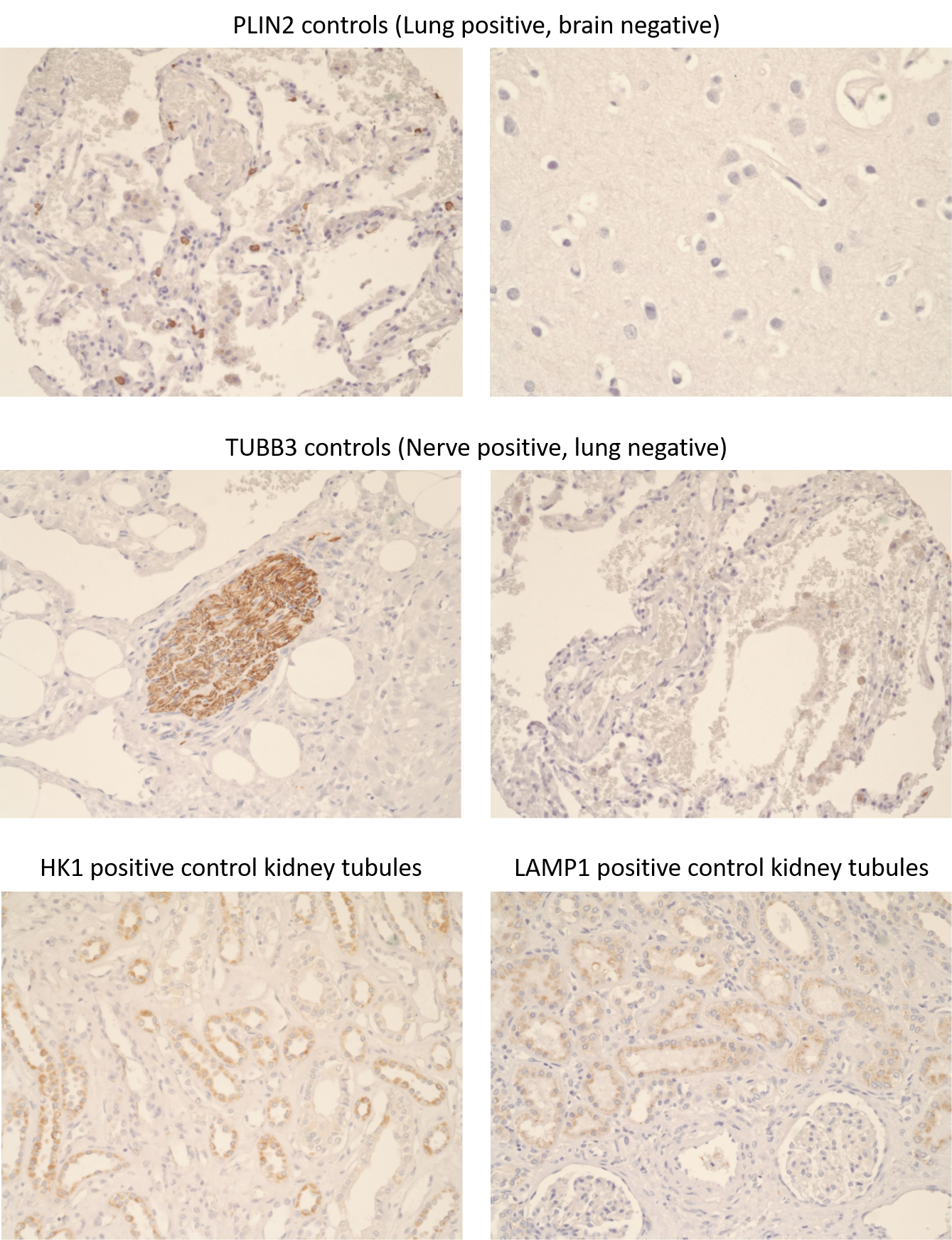

Supplement: Supplementary file 3 — Additional file 3: Table S2. Details of antibodies used for validation immunohistochemistry. [file 12916_2021_2071_MOESM3_ESM.png]

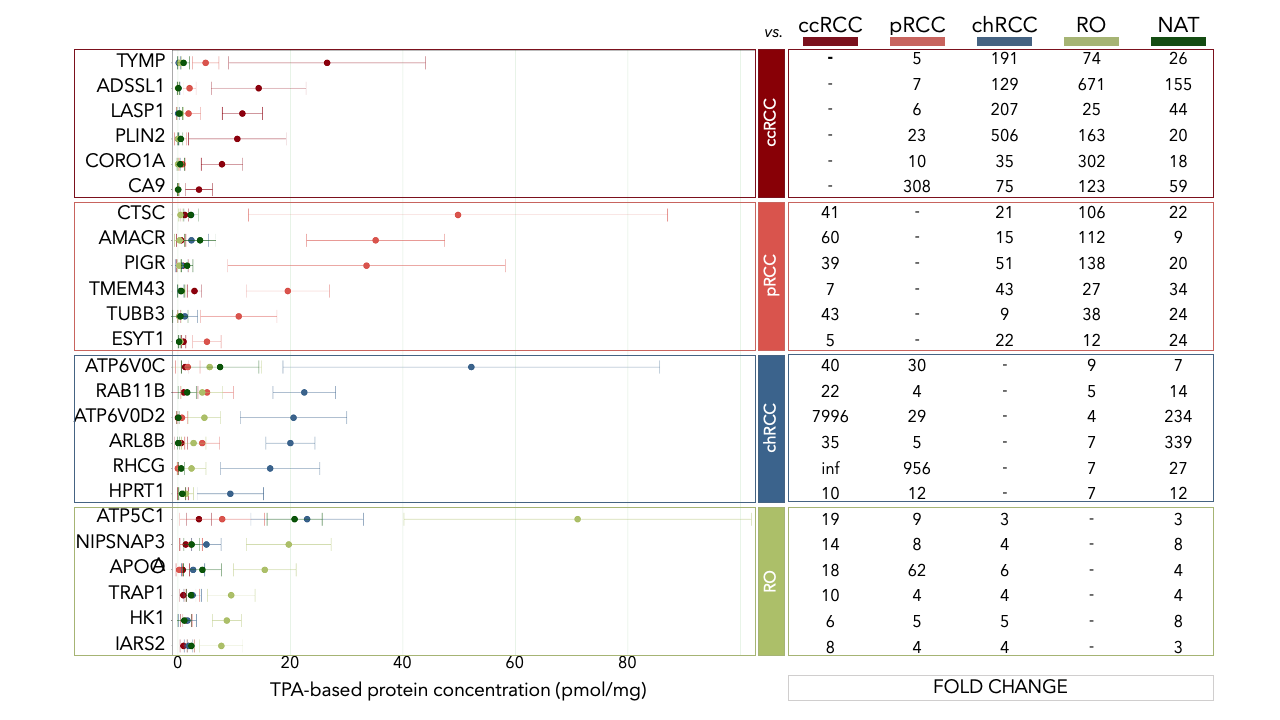

Supplement: Supplementary file 7 — Additional file 7: Fig. S2. TPA-based concentrations of the proteins with the highest differential expression between tissue biopsies (fold change) for each subtype. Clear cell renal cell carcinoma (ccRCC, FC ≥ 5); papillary renal cell carcinoma (pRCC, FC ≥ 9); chromophobe renal cell carcinoma (chRCC, FC ≥ 4); renal oncocytoma (RO, FC ≥ 3). [file 12916_2021_2071_MOESM7_ESM.png]

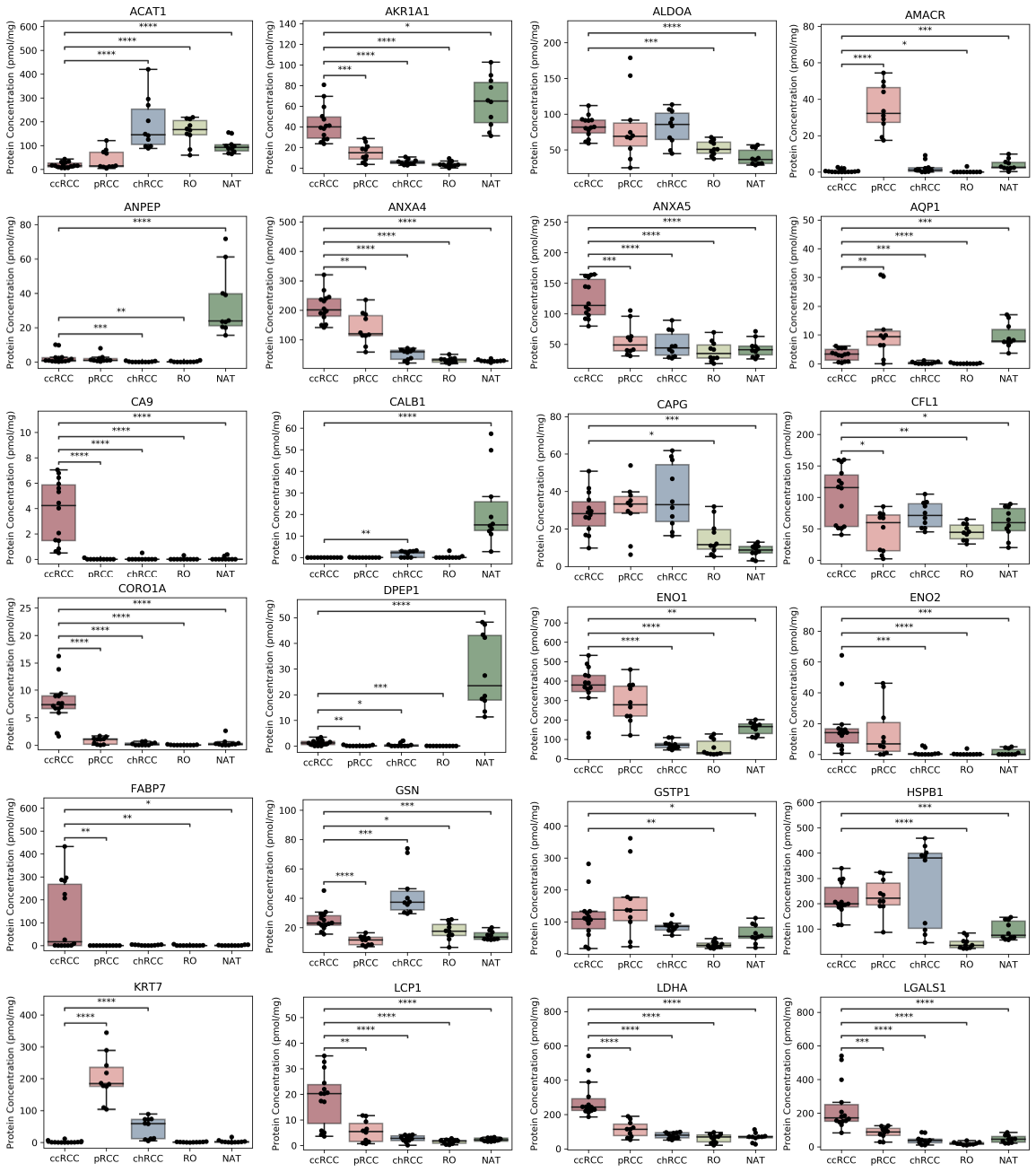

Supplement: Supplementary file 8 — Additional file 8: Fig. S3. TPA concentration values of proteins described in literature - Part I. Statistical analysis was performed using pairwise Mann Whitney test (*p ≤ 0.005; **p ≤ 0.001; ***p ≤ 0.0001; ****p ≤ 0.00001). [file 12916_2021_2071_MOESM8_ESM.png]

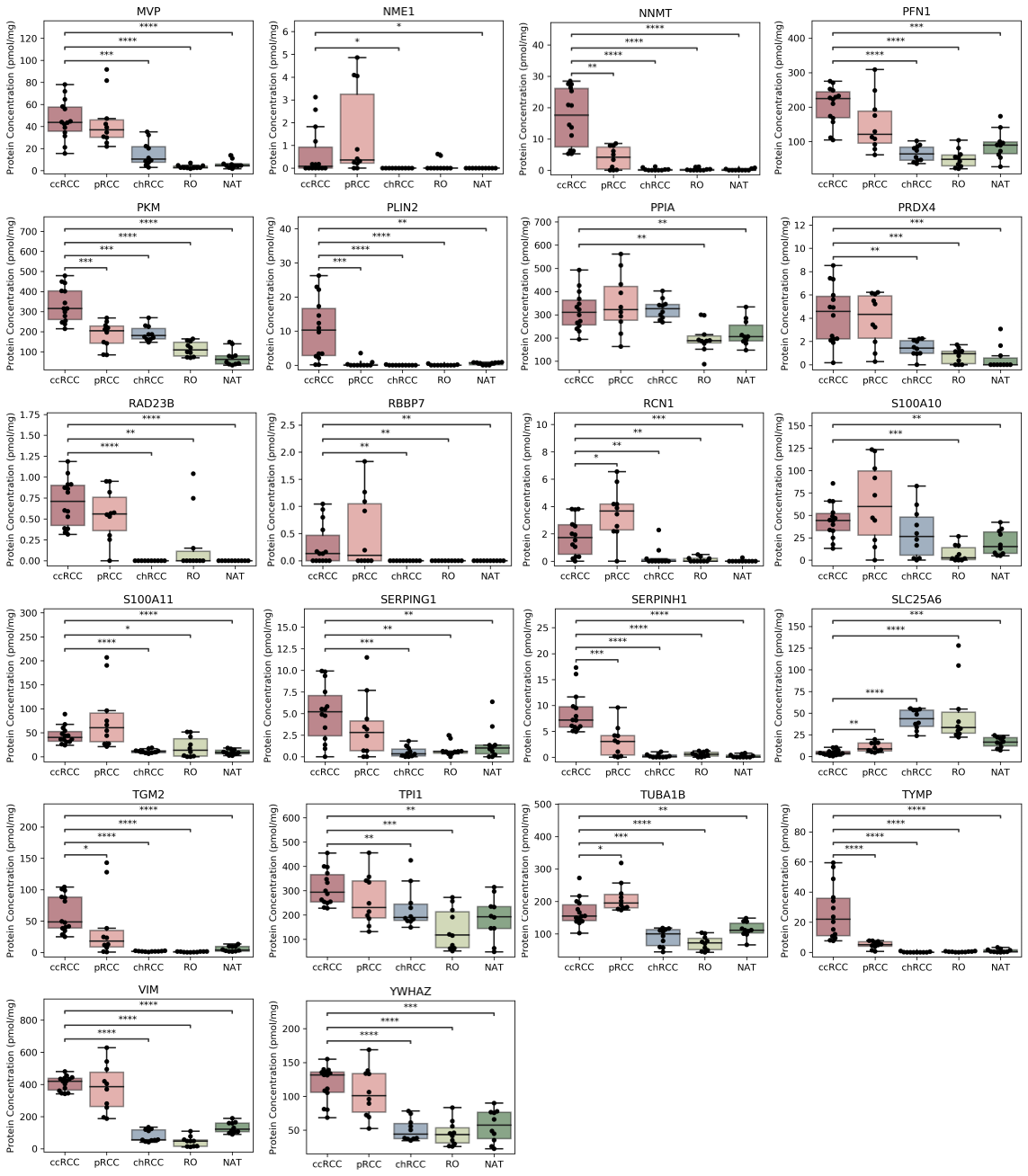

Supplement: Supplementary file 9 — Additional file 9: Fig. S4. TPA concentration values of proteins described in literature - Part II. Statistical analysis was performed using pairwise Mann Whitney test (*p ≤ 0.005; **p ≤ 0.001; ***p ≤ 0.0001; ****p ≤ 0.00001). [file 12916_2021_2071_MOESM9_ESM.png]
