# Supplementary material for: Absolute quantitative proteomics using the total protein approach to identify novel clinical immunohistochemical markers in renal neoplasms
Source: BMC Med. 2021 Sep 6;19:196. doi: 10.1186/s12916-021-02071-9 (PMC8420025; doi:10.1186/s12916-021-02071-9)
Supplement: Supplementary file 4 — Additional file 4: Table S3. Sensitivity, specificity, positive predictive values, and negative predictive values calculated for IHC biomarkers. [file 12916_2021_2071_MOESM4_ESM.docx]

**Table S3.** Sensitivity, specificity, positive predictive values and negative predictive values calculated for IHC biomarkers.

|  | **CCRCC** | **Non-CCRCC** | **Total** |
| --- | --- | --- | --- |
| **PLIN-2 2+/3+** | 36 | 0 | 36 |
| **PLIN-2 0/1+** | 4 | 68 | 72 |
| **Total** | 40 | 68 | 108 |

Sensitivity = TP / (TP+FN) = 36 / (36+4) = 36/40 = 90%

Specificity = TN / (TN+FP) = 68 / (68+0) = 68/68 = 100%

Positive predictive value = TP / (TP+FP) = 36 / (36+0) = 36/36 = 100%

Negative predictive value = TN / (TN+FN) = 68 / (68+4) = 68/72 = 94.4%

|  | **pRCC** | **Non-pRCC** | **Total** |
| --- | --- | --- | --- |
| **TUBB3 2+/3+** | 14 | 0 | 14 |
| **TUBB3 0/1+** | 12 | 82 | 94 |
| **Total** | 26 | 82 | 108 |

Sensitivity = TP / (TP+FN) = 14 / (14+12) = 14/26 = 53.8%

Specificity = TN / (TN+FP) = 82 / (82+0) = 82/82 = 100%

Positive predictive value = TP / (TP+FP) = 14 / (14+0) = 14/14 = 100%

Negative predictive value = TN / (TN+FN) = 82 / (82+12) = 82/94 = 87.2%

|  | **RO** | **Non-RO** | **Total** |
| --- | --- | --- | --- |
| **HK1 2+/3+ and >90% of cells** | 29 | 1 | 30 |
| **HK1 0/1+ or <90% of cells** | 1 | 77 | 78 |
| **Total** | 30 | 78 | 108 |

Sensitivity = TP / (TP+FN) = 29 / (29+1) = 29/30 = 96.7%

Specificity = TN / (TN+FP) = 77 / (77+1) = 77/78 = 98.7%

Positive predictive value = TP / (TP+FP) = 29 / (29+1) = 29/30 = 96.7%

Negative predictive value = TN / (TN+FN) = 77 / (77+1) = 77/78 = 98.7%

|  | chRCC | Non-chRCC | Total |
| --- | --- | --- | --- |
| LAMP1 Diffuse | 11 | 0 | 11 |
| LAMP1 Apical/Focal/Negative | 1 | 96 | 97 |
| Total | 12 | 96 | 108 |

Sensitivity = TP / (TP+FN) = 11 / (11+1) = 11/12 = 91.7%

Specificity = TN / (TN+FP) = 96 / (96+0) = 96/96 = 100%

Positive predictive value = TP / (TP+FP) = 11 / (11+0) = 11/11 = 100%

Negative predictive value = TN / (TN+FN) = 96 / (96+1) = 96/97 = 99%
