# Supplementary material for: Absolute quantitative proteomics using the total protein approach to identify novel clinical immunohistochemical markers in renal neoplasms
Source: BMC Med. 2021 Sep 6;19:196. doi: 10.1186/s12916-021-02071-9 (PMC8420025; doi:10.1186/s12916-021-02071-9)
Supplement: Supplementary file 10 — Additional file 10: Table S6. Current available IHC markers for the diagnosis of renal neoplasms. [file 12916_2021_2071_MOESM10_ESM.docx]

**Table S6.** Immunohistochemistry in the differential diagnosis of renal neoplasms.

| **IHC** | **ccRCC** | **pRCC** | **chRCC** | **RO** | **ccpRCC** | **t-RCC** | **HLRCC** | **SDH deficient RCC** | **ACD, RCC** | **AML** |
| --- | --- | --- | --- | --- | --- | --- | --- | --- | --- | --- |
| **PAX 8** | + | + | + | + | + | + | + | + | + | - |
| **CA IX** | + (diffuse) | - | - | - | +  (cup-like) | -/focal | - | - | - | - |
| **CK7** | - | +/- | +  (diffuse) | +  (scattered single cells) | + | - | - | - | - | - |
| **AMACR** | -/+ | + | - | - | - | +/- | - | - | + | - |
| **C-kit** | - | - |  | + | + | -/+ | - | - | - | - |
| **VIM** | + | +/- | - | - | - | + | N/A | N/A | N/A | + |
| **Cathepsin K** | - | - | - | - | - | +/- | - | - | - | + |
| **Melanocytic markers** | - | - | - | - | - | +/- | - | - | - | + |
| **Others** | None | None | None | None | None | TFE-3 and TFE-B | FH loss, 2SC+ | SDHB loss | None | SMA+, Calponin+ |

ACD RCC: acquired cystic disease associated renal cell carcinoma; AMACR: alpha methyl acyl co-A racemase; AML: angiomyolipoma; CK: cytokeratin; ccRCC: Clear cell renal cell carcinoma; chRCC: chromophobe renal cell carcinoma; FH: Fumerate hydratase; HLRCC: hereditary leiomyomatosis associated renal cell carcinoma; N/A: not applicable or not available; pRCC: Papillary renal cell carcinoma; PAX: Paired box; RO: renal oncocytoma; SMA: smooth muscle actin

SDH deficient RCC: Succinate dehydrogenase deficient renal cell carcinoma; t-RCC: translocation associated renal cell carcinoma; TFE-3: transcription factor enhancer 3.
